# Rheological and Physicochemical Characterization of Structured Chia Oil: A Novel Approach Using a Low-Content Shellac Wax/Beeswax Blend as Oleogelant

**DOI:** 10.3390/gels11090680

**Published:** 2025-08-25

**Authors:** Eduardo Morales, Katerine Marilaf, Mónica Rubilar, Ingrid Contardo, Marcela Quilaqueo, Sonia Millao, Mariela Bustamante, César Burgos-Díaz, Karla Garrido-Miranda

**Affiliations:** 1 Scientific and Technological Bioresource Nucleus, BIOREN, Universidad de La Frontera, Avenida Francisco Salazar 01145, Temuco 4811230, Chile; monica.rubilar@ufrontera.cl (M.R.); marcela.quilaqueo@ufrontera.cl (M.Q.); sonia.millao@ufrontera.cl (S.M.); mariela.bustamante@ufrontera.cl (M.B.); karla.garrido@ufrontera.cl (K.G.-M.); 2 Department of Chemical Engineering, Faculty of Engineering and Science, Universidad de La Frontera, Temuco 4811230, Chile; k.marilaf01@ufromail.cl; 3 Biopolymer Research & Engineering Laboratory (BiopREL), School of Nutrition and Dietetics, Faculty of Medicine, Universidad de los Andes, Chile, Monseñor Álvaro del Portillo 12.455, Las Condes, Santiago 7620086, Chile; icontardo@uandes.cl; 4 Centro de Investigación e Innovación Biomédica (CIIB), Universidad de los Andes, Chile, Monseñor Álvaro del Portillo 12.455, Las Condes, Santiago 7620086, Chile; 5 Agriaquaculture Nutritional Genomic Center, CGNA, Temuco 4780000, Chile; cesar.burgos@cgna.cl

**Keywords:** oleogelant, shellac wax, beeswax, blend, structured chia oil

## Abstract

Vegetable oils structured with natural wax blends have attracted increasing interest due to their tunable crystallization and gelling behavior. This study evaluated the structuring of chia oil (ChO) using low concentrations (1–5%) of a shellac wax (SW) and beeswax (BW) blend in a 1:1 ratio, focusing on physicochemical, viscoelastic, and thixotropic properties. ChO structured with 1% SW/BW formed a weak network with high oil loss, whereas concentrations of 3–5% formed denser networks, resulting in OBC values of 75.6–88.4% and firmness values of 16.9–55.1 g. Structuring with 5% SW/BW significantly reduced peroxide values (*p* < 0.05), indicating a reduction in oxidative deterioration after oleogelation, while concentrations of 1–3% had no significant effect (*p* > 0.05). Although induction periods were slightly extended in structured samples, differences across oleogelant concentrations were not statistically significant (*p* > 0.05). Rheological analysis revealed that 3–5% SW/BW-structured ChO exhibited semisolid gel behavior, characterized by enhanced deformation resistance and thermal stability. Thixotropic recovery tests revealed that structural recovery improved as the deformation amplitude decreased within the linear viscoelastic range, suggesting that thixotropic behavior was influenced by oleogelant concentration. These findings demonstrate the potential of SW/BW-structured ChO as fat alternatives in lipid-based foods that require mechanical resilience, structural recovery, and enhanced oxidative stability, even at low wax levels.

## 1. Introduction

The extensive use of animal fats and hydrogenated vegetable oils (solid structures) in the food industry is attributed to their significant role in determining the texture of food products and their high oxidative stability [1]. These fats are widely used in the bakery and confectionery industries, typically containing substantial amounts of saturated fatty acids (SFA) and/or trans fatty acids (TFA). However, excessive intake of SFA and TFA has been associated with an increased risk of obesity, diabetes, and coronary heart disease [2]. Replacing industrially produced TFA with vegetable oils rich in polyunsaturated fatty acids (PUFA) has reduced cardiovascular disease risk [3]. For example, chia oil (ChO) is notable for its high PUFA content (78.21 g/100 g), with 60.04 g/100 g consisting of α-linolenic acid (omega-3) [4]. However, the direct replacement of solid fats with vegetable oils in food formulations can compromise key quality attributes such as texture, mouthfeel, and oxidative stability [5]. To address this, oleogelation has emerged as a practical approach for structuring vegetable oils into solid or semisolid matrices, offering a promising alternative to traditional solid fats in the production of lipid-based foods [6,7]. Structured oils are created by entrapping liquid oil within a three-dimensional network formed by one or more oleogelators [8]. Due to their availability and gelling ability, vegetable waxes have emerged as effective oleogelators for structuring liquid oils [9]. These natural waxes have attracted considerable interest in food science because they can form gels at low concentrations (as low as 0.5%), are relatively inexpensive, and are approved as food additives [8,10,11]. In particular, shellac wax (SW) and beeswax (BW) have been extensively used both for oil structuring and emulsion stabilization [12,13]. Since these waxes have distinct chemical compositions, combining them may compensate for their limitations and expand their range of applications in food formulations [11,14].

SW is a natural polymer derived from the resinous secretion of the insect *Laccifer lacca*, found mainly in India, Burma, Thailand, and southern China [15]. It is recognized as safe (GRAS) by the United States Food and Drug Administration (FDA), allowing its use as a food additive and as a raw material in edible coatings and film formulations [9]. This polymer has a melting point between 75 and 85 °C and is used in concentrations from 1 to 6% to structure oils and stabilize emulsions [12,15,16]. Similarly, BW is a well-known oleogelant for structuring edible vegetable oils due to its ability to gel at concentrations ranging from 1 to 10% with a melting point around 65–70 °C [3,13,17,18]. It is also approved by the FDA for food use [19]. The linear crystalline structure of BW mimics the solid fat crystal network, which significantly influences the texture and plasticity of structured oils [18]. It also enhances the stability of water-in-oil (W/O) emulsions and exhibits notable thixotropic properties [13].

Wax-based blends have increasingly been used to structure oils in lipid-based food systems. These blends have drawn significant attention in recent years, as adjusting the proportions between the two oleogelators can modify their crystallization and gelation behavior [11,20]. Moreover, several studies have demonstrated that combining two different waxes or wax with another type of oleogelator can reduce the total concentration of structuring agents required while preserving their gelling properties [21,22]. For example, combinations such as β-sitosterol/BW (10–20%) in sunflower oil [23], BW/SW (10%) in linseed and canola oil [24], BW/stearic acid (5–15%) in sesame and rice oils [25], candelilla wax/BW (5%) in soybean oil [6], and BW/glycerol monostearate (5–10%) in Tenebrio molitor larvae oil [22] have all shown synergistic effects that alter the structural framework and rheological properties of the resulting gels. Nevertheless, the required concentrations often remain relatively high even when combining different structuring agents. Moreover, several studies have reported that wax-based oleogels, when used at high concentrations (generally above 5%), can produce a waxy mouthfeel along with undesirable flavours or odours, which may limit their applicability in food products [26].

Considering the above, this study proposes a novel approach to structuring chia oil (ChO) using low concentrations (≤5%) of a natural wax blend composed of SW and BW. To our knowledge, only a few studies have formulated oleogels with ChO using natural waxes as oleogelants. Moreover, in contrast to previous studies, which typically required higher levels of oleogelators, this work demonstrates that ChO can be effectively structured with lower wax concentrations while maintaining a high oil binding capacity; a semisolid, gel-like texture; resistance to lipid oxidation; and the ability to recover its structure after undergoing a full deformation sweep for future food applications. Additionally, this study aimed to evaluate the physicochemical, viscoelastic, and thixotropic characteristics of ChO structured with low-SW/BW blend concentrations. The results of this study will contribute to a deeper understanding of the behaviour of natural wax blends at low concentrations as structuring agents in vegetable oils. Furthermore, they will highlight their potential as sustainable alternatives for the development of food products that undergo mechanical processing, such as mixing, blending, or shearing.

## 2. Results and Discussion

### 2.1. Physicochemical Properties of Structured ChO

#### 2.1.1. Oil Binding Capacity, Firmness and Microstructure

Oil binding capacity (OBC) is a key parameter in structured vegetable oils, as it reflects the physical retention of liquid oil within the three-dimensional network formed by the oleogelant. OBC represents the proportion of oil effectively retained in the gel matrix [27]. Table 1 shows the OBC values of ChO structured with the SW/BW blend and individual SW and BW waxes as controls at 1, 3, and 5% concentrations.

**Figure 1:**
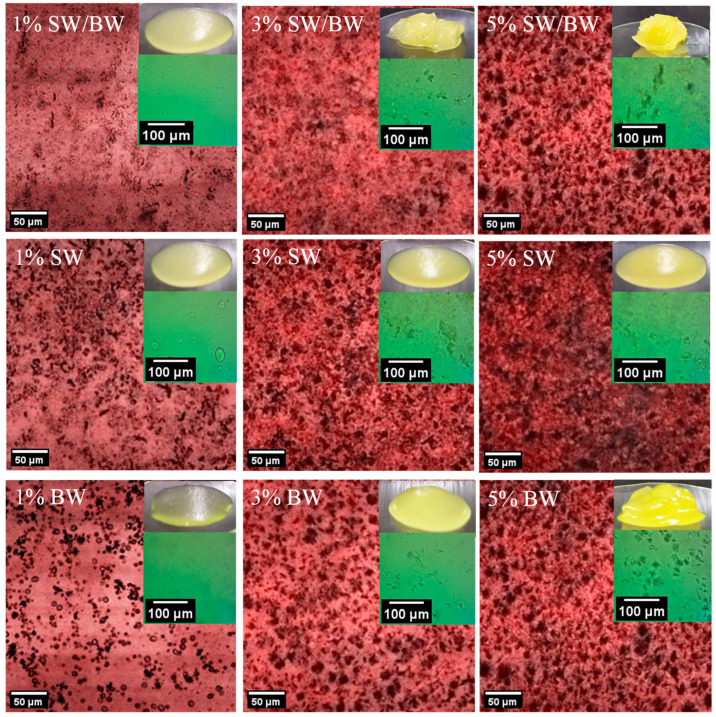
Confocal microscopy and optical microscopy images in ChO structured with SW/BW, SW, and BW at 1, 3, and 5% oleogelant.

The results indicated that ChO structured with 1% oleogelant in the blend and controls had OBC values below 50%, indicating a weak structure and high oil migration (SW/BW: 43.6 ± 1.23% OBC and 56.4% oil loss; SW: 39.8 ± 0.71% OBC and 60.2% oil loss; BW: 10.0 ± 1.24% OBC and 90.0% oil loss). Nevertheless, ChO structured with 3% oleogelant showed a significant increase (*p* < 0.05) in OBC, with values above 65%, and reduced oil migration compared to formulations containing only 1% structuring agent, exhibiting a semisolid structure. In particular, the SW/BW blend showed an OBC of 75.6 ± 1.12% and an oil loss of 24.4%; SW wax showed a OBC of 67.3 ± 0.29% and a loss of 32.7%; and BW wax showed a OBC of 66.3 ± 1.04% with a loss of 33.7%. In addition, a slight increase in the OBC value was observed for the SW/BW blend at 3% compared to the individual controls, which could be attributed to possible interactions between the two waxes that would modify the crystallization behaviour and microstructure of the network formed, thus improving its oil retention capacity [11]. This trend is consistent with findings by Jeong and Oh [22], who observed increased OBC with higher concentrations of a BW/glycerol monostearate blend in structuring Tenebrio molitor larval oil. The ChO structured with a 5% concentration of the SW/BW blend, as well as with individual waxes (SW and BW), exhibited a significant increase in OBC values (*p* < 0.05). However, no statistically significant differences (*p* > 0.05) were observed between the blend and the individual waxes. Specifically, the OBC values were 88.4 ± 1.01% for the SW/BW blend, 87.6 ± 1.65% for SW, and 91.3 ± 0.53% for BW, corresponding to oil losses of 11.6%, 12.4%, and 8.7%, respectively. These results indicate that the crystalline network formed in the structured ChO at 5% concentration created a more homogeneous physical barrier compared to oleogels prepared at 1 and 3%, as confirmed by confocal microscopy (Figure 1). Overall, increasing the oleogelant concentration enhanced the stability of the structured ChO by reducing oil loss, limiting the mobility of the liquid phase, and thereby promoting the formation of a more stable oleogel [22,28].

On the other hand, preliminary studies revealed that after the ChO structuring process with 1% of BW/SW, SW, and BW, no firmness values were detected since they were lower than the minimum detection value by the firmness analyser (<0.5 g). These results coincide with the low OBC observed when ChO was structured with 1% of the blend and in the controls. This behaviour is attributed to the limited crystal network formation within the oleogel structure [11]. Subsequently, the results showed that the firmness value of the SW/BW (16.9 ± 0.37 to 55.1 ± 4.15 g), SW (9.59 ± 0.95 to 31.6 ± 1.91 g), and BW (46.6 ± 2.89 to 134.8 ± 6.37 g) increased significantly (*p* < 0.05) when the content of the oleogelants increased from 3 to 5%, presenting structured oils with a semisolid structure comparable to a gel in every case. The results also revealed that the firmness value of the structured ChO prepared with SW/BW was significantly higher (*p* < 0.05) than with SW. However, the firmness values of BW were significantly higher (*p* < 0.05) than the blend. Several studies have reported that increasing the concentration of oleogelant agent increases the firmness of structured oils with waxes and vegetable oils due to the growth of the solid volume fraction in dense networks that effectively retain the surrounding liquid oil [29,30]. Our study demonstrated that using a 3% concentration of oleogelant was sufficient to obtain a firm gel with semisolid characteristics.

Figure 1 shows the microstructure of ChO structured with SW/BW, SW, and BW at different concentrations of structuring agent (1%, 3%, and 5%), observed by scanning confocal microscopy. Aggregates (dark areas) were observed in all three types of structured ChO, whose distribution depended on the concentration of the oleogelant. In addition, optical microscopy images can be seen in the upper right corner. Previous studies have described the presence of platelet-shaped crystals in wax-structured oils, which can occur as discrete or aggregated structures [11,31]. The results showed that ChO structured with 1% oleogelant exhibited more dispersed crystals than formulations containing higher concentrations of the structuring agent, consistent with the low OBC values (Table 1). However, no substantial differences were detected in the distribution or size of the crystals between the formulations with 3 and 5% oleogelant in the SW/BW blend and the individual waxes, as can be seen under optical microscopy. These formulations’ higher crystalline aggregation density favoured better oil immobilization in the ChO. According to Fayaz et al. [32], oils structured with waxes tend to form platelet-like crystals due to van der Waals interactions, which allow the immobilization of liquid oil within a three-dimensional network. This information was corroborated through FTIR spectra (Figure S1, Supplementary Material), where the characteristic bands of ChO were observed in the different oleogels: the peaks at 2923 cm^−1^ and 2853 cm^−1^, corresponding to the asymmetric stretching of CH from methyl groups and the symmetric stretching of the CH methylene group, respectively, associated with saturated fatty acids [33]. The subsequent peak at 1742 cm^−1^ is attributed to the stretching of the C=O carbonyl group from ester groups present in lipids and fatty acids of ChO [34]. Lastly, the peaks at 1460 cm^−1^ and 1159 cm^−1^ correspond to the asymmetric deformation of the methylene group and the out-of-plane deformation of the methylene group present in lipids [35]. The characteristic spectra of BW and SW are also shown in Figure S1. These waxes had almost similar FTIR peaks, such as at 2914 cm^−1^ and 2846 cm^−1^ corresponding to C–H stretching, at 1463 cm^−1^ associated with C–H bending or scissoring, at 1166 cm^−1^ attributed to the COC stretching, and at 719 cm^−1^ corresponding to C–H rocking,;all these peaks associated with the hydrocarbon chains of the waxes [36]. The oleogels maintain the characteristic ChO bands, indicating that no new covalent bonds were formed. Therefore, only weak interactions, such as van der Waals forces or hydrogen bonds, are present.

#### 2.1.2. Peroxide Value and Induction Period

The peroxide value (PV) and induction period (IP at 90 °C) were used as indicators of the oxidation state of chia oil (ChO) structured with the SW/BW blend and the controls (SW and BW) after the oleogelation process at 82 °C. For comparison purposes, unstructured ChO was also subjected to the same temperature used during oleogelation (Table 1). ChO is known to have a high proportion of unsaturated fatty acids susceptible to oxidation. Hence, exposure to high temperatures during oleogelation contributes to increased lipid oxidation. Thus, in the initial oxidation phase, fatty acids react with oxygen to form odourless primary compounds, such as peroxides [31]. The results showed that unstructured ChO, heated to the oleogelation temperature (82 °C), exhibited a significant increase in PV (*p* < 0.05) compared to ChO structured with the SW/BW blend and BW at 5%. This difference may be attributed to the higher oil retention capacity of the structured gels, which increased with oleogelant concentration, as evidenced by the OBC results. These findings highlight the critical role of oleogelant content in the gel network for inhibiting oil oxidation, as more compact structures effectively restrict oxygen penetration and diffusion within the samples, thereby slowing the oxidation rate [4,24]. Conversely, when the oleogelation process was conducted with 1 and 3% oleogelant concentrations for both the blend and its controls, no significant differences (*p* > 0.05) in PV were observed compared to the unstructured ChO. Notably, after the oleogelation process, the PVs of all structured ChO samples, regardless of the oleogelant type or concentration used, remained well below the maximum limit established for cold-pressed oils (15 meq O_2_/kg sample [37]).

On the other hand, the oxidative stability of the samples was evaluated using the direct oxygen consumption method with the Oxitest reactor. Oxidative stability was expressed as the induction period (IP), defined as the time required for a sharp increase in the rate of lipid oxidation to occur [38]. The results showed that the ChO structured with the SW/BW blend exhibited a longer IP at all concentrations tested compared to the unstructured oil. However, no significant differences (*p* > 0.05) were observed between the different levels of the oleogelant. Notably, ChO structured with 5% BW presented a significant increase (*p* < 0.05) in IP compared to the unstructured oil, indicating that the oleogel delayed the formation of secondary oxidation products and thereby enhanced the oxidative stability of ChO [2]. In contrast, ChO structured with 1% and 3% SW exhibited lower IP values than the unstructured oil, suggesting either a lack of protective effect from the structuring agent or the presence of pro-oxidant compounds in these formulations. Hwang et al. [39] have emphasized that this pro-oxidant effect is not related to hydrocarbons or wax esters but rather to the presence of minor components, especially free fatty acids, which are well known to play a pro-oxidant role in oils [38].

### 2.2. Rheological Properties of Structured ChO

#### 2.2.1. Shear Sweep

**Figure 2:**
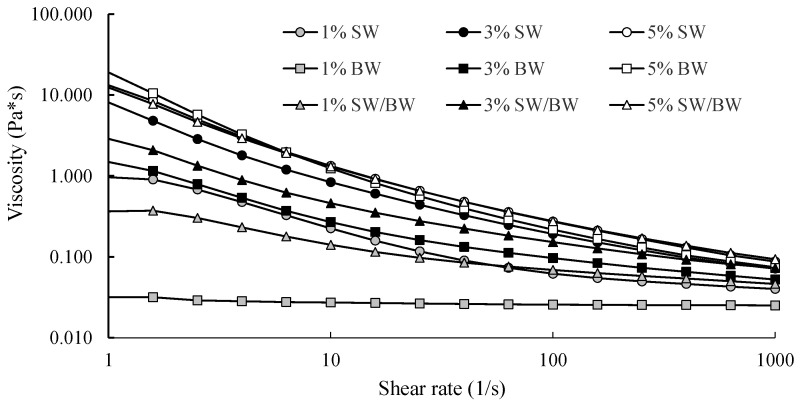
Apparent viscosity (Pa.s) in ChO structured with SW/BW, SW, and BW at 1, 3, and 5% oleogelant, with a shear rate ranging from 1 to 1000 (1/s).

Viscosity measurements of structured ChO demonstrated typical pseudoplastic fluid behaviour, with apparent viscosity decreasing as the shear rate increased (Figure 2). The results showed that ChO structured with 1 and 3% SW exhibited remarkably higher viscosity than those structured with BW or the SW/BW blend, indicating greater resistance to flow. These differences can be attributed to variations in molecular interactions and the types of crystals formed at low concentrations: SW promotes the formation of spherulitic crystals, whereas BW leads to the development of needle-like crystals within the structured oil [40]. Although notable differences in viscosity were observed among SW-, BW-, and SW/BW-structured ChO at lower concentrations, this trend did not persist when the oleogelant concentration reached 5%. At this level, all samples exhibited similar viscosity profiles across the entire range of shear rates. This behaviour suggests that, as confirmed by microscopy (Figure 1), increasing the concentration of SW, BW, or their blend promotes the formation of more extensive crystalline networks, thereby enhancing shear stability [18].

#### 2.2.2. Strain Sweep

**Figure 3:**
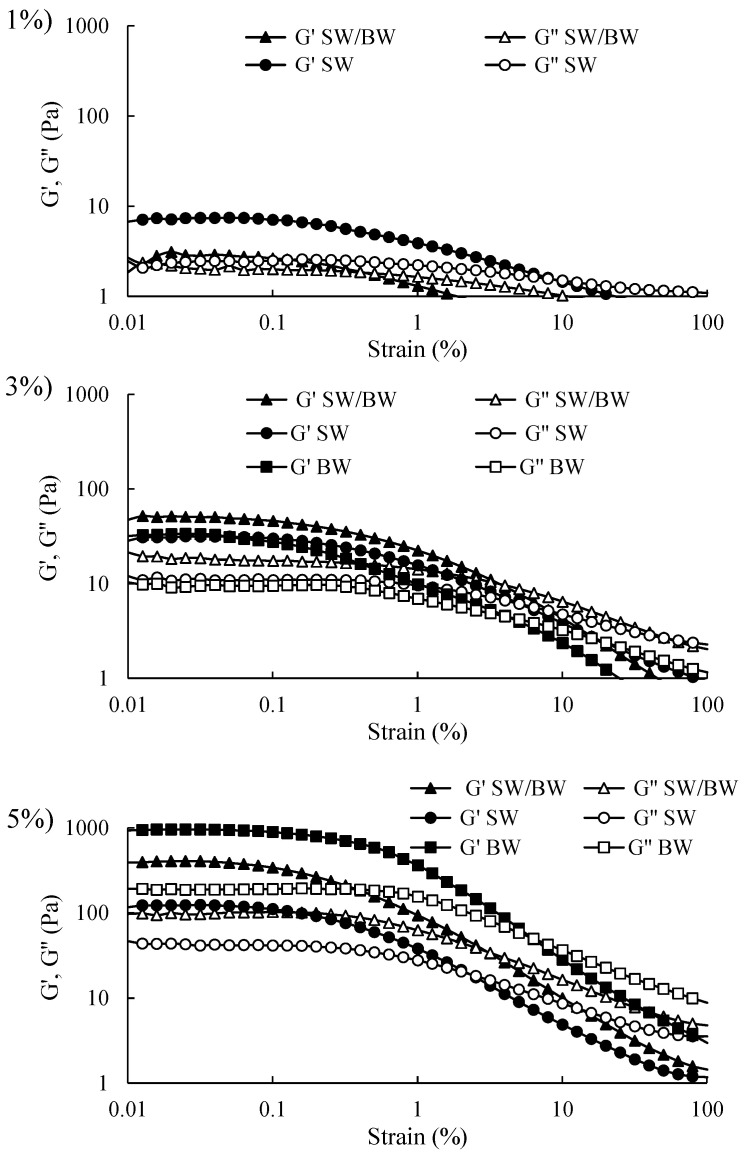
Strain sweep in ChO structured with SW/BW, SW, and BW at 1, 3, and 5% oleogelant.

Viscoelastic tests are highly valuable and yield meaningful insights within the linear viscoelastic region (LVR). Accordingly, the initial step in oscillatory rheological testing involves determining the LVR using a strain sweep test to assess the strength of the elastic properties of structured oils. It is important to note that the storage modulus (G′) represents the elastic component and reflects the solid-like behaviour of a sample. In contrast, the loss modulus (G″) represents the viscous component and reflects liquid-like properties [2]. As illustrated in Figure 3, strain sweep rheograms can be divided into two distinct regions: the LVR, where both G′ and G″ remain approximately constant, and the non-linear region, where G′ and G″ begin to decrease with increasing deformation (considerable strain). Within the non-linear region, as strain increases, G′ and G″ intersect at the crossover point (G′ = G″). This point signifies the material’s internal structure breakdown, leading to flow as G″ surpasses G′. The results demonstrated that G′ exceeded G″ before the crossover point for structured oil samples at varying concentrations, indicating solid-like elastic behaviour within the LVR. Structured ChO sharply declined in G′ and G″ at strains above 1%. Conversely, BW/SW and BW prepared at 3% and 5% concentrations displayed higher G′ values than SW, suggesting that the SW/BW blend and BW enhanced gel strength, likely through increased intermolecular chain entanglements. In contrast, all structured ChO samples at 1% concentration exhibited a smaller LVR and lower G′ values within the LVR than at higher concentrations, indicating weak gel characteristics, which store less energy, and this storage capacity decreases even further with greater deformation. These results are consistent with the firmness results (Table 1) and microstructure (Figure 1), where 1% oleogelant concentration did not generate sufficient cross-linking to offer resistance to compression, which was attributed to the limited crystal network formation within the oleogel structure. These samples exhibited a greater degree of flow at a shear rate ranging from 1 to 1000 (1/s) (Figure 2), dissipated more energy (Figure 3), and did not easily recover their original shape after a large deformation (100% strain). The observed enhancement in gel strength with SW/BW in oil structuring, relative to SW, may be attributed to robust reciprocal interactions among SW and BW within the crystalline lattice. These interactions likely contribute to developing a more compact, three-dimensional network with superior gel properties [29]. Therefore, compared to SW, the 5% SW/BW and BW samples exhibited more substantial elastic properties and demonstrated a gel character with higher structural resistance to deformation, indicating that more energy can be stored in BW samples at 5% oleogelant concentration (Figure 3), which generates more elastic and firmer gels (Table 1) than SW, its mixture, or lower concentrations of oleogelant, flowing less (Figure 2) but easily recovering its original shape. Possibly, the greater firmness, elasticity, and deformability properties of these samples made with BW compared to SW or their mixtures can be attributed to the greater presence of monoesters in BW (approximately 48.0–86.0%), which has been pointed out as one of the factors responsible for increasing the firmness of the oleogels [41].

#### 2.2.3. Frequency Sweep

**Figure 4:**
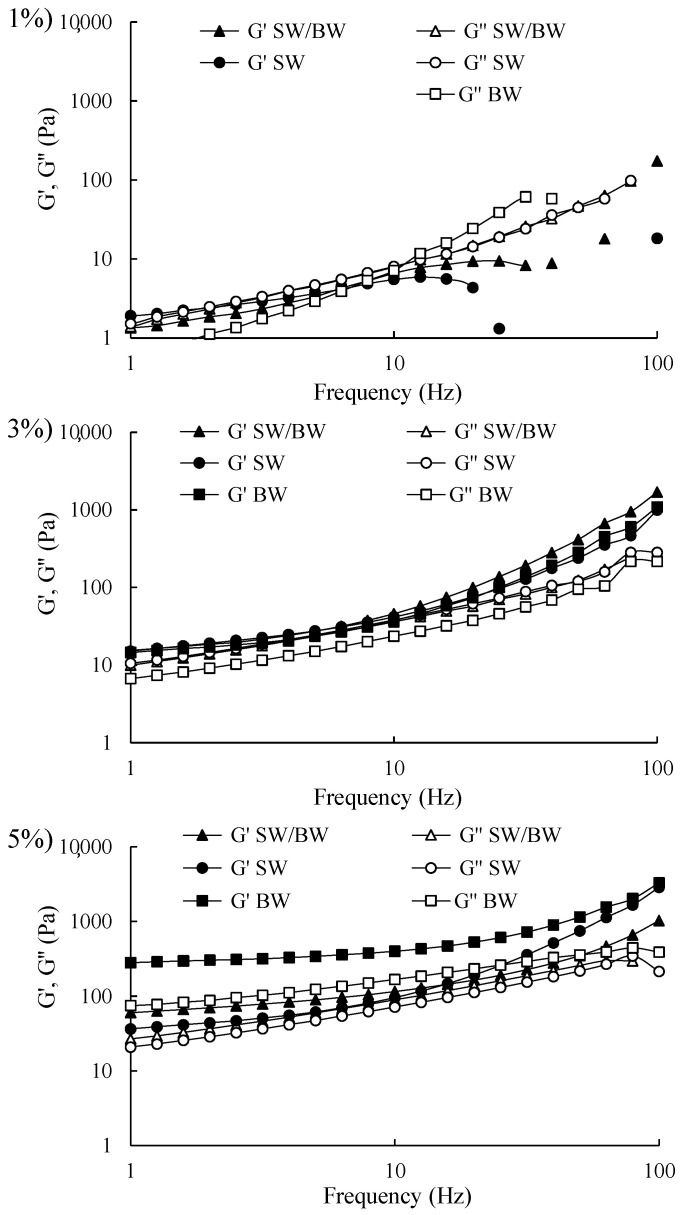
Frequency sweep in ChO structured with SW/BW, SW, and BW at 1, 3, and 5% oleogelant.

Frequency sweep rheograms were conducted within the linear viscoelastic region (LVR) of structured ChO at concentrations of 1, 3, and 5% to provide insights into the strength of their internal gel structures, as illustrated in Figure 4. The structured ChO prepared at 3% and 5% exhibited similar behaviour, with storage modulus (G′) values exceeding loss modulus (G″) values across the entire frequency range (1–100 Hz), indicating a solid-like behaviour comparable to a gel. Notably, no crossover point between G′ and G″ (where G′ = G″) was observed, suggesting that the structured ChO at 3% and 5% remained relatively stable over the frequency range, implying adequate stability during storage [42]. However, the 5% BW demonstrated significantly higher G′ values than the SW/BW, with both structured ChO displaying higher G′ values than SW. This behaviour suggests greater resistance to deformation due to the formation of more robust gel networks composed of wax crystal fragments [2]. These findings indicate that the structured ChO can withstand oscillatory stress within the tested frequency range, maintaining their semisolid properties without structural breakdown under high-frequency oscillations [22,43]. In contrast, the 1% structured ChO exhibited G′ values lower than G″ throughout the frequency range, indicating a liquid-like behaviour.

#### 2.2.4. Temperature Sweep

**Figure 5:**
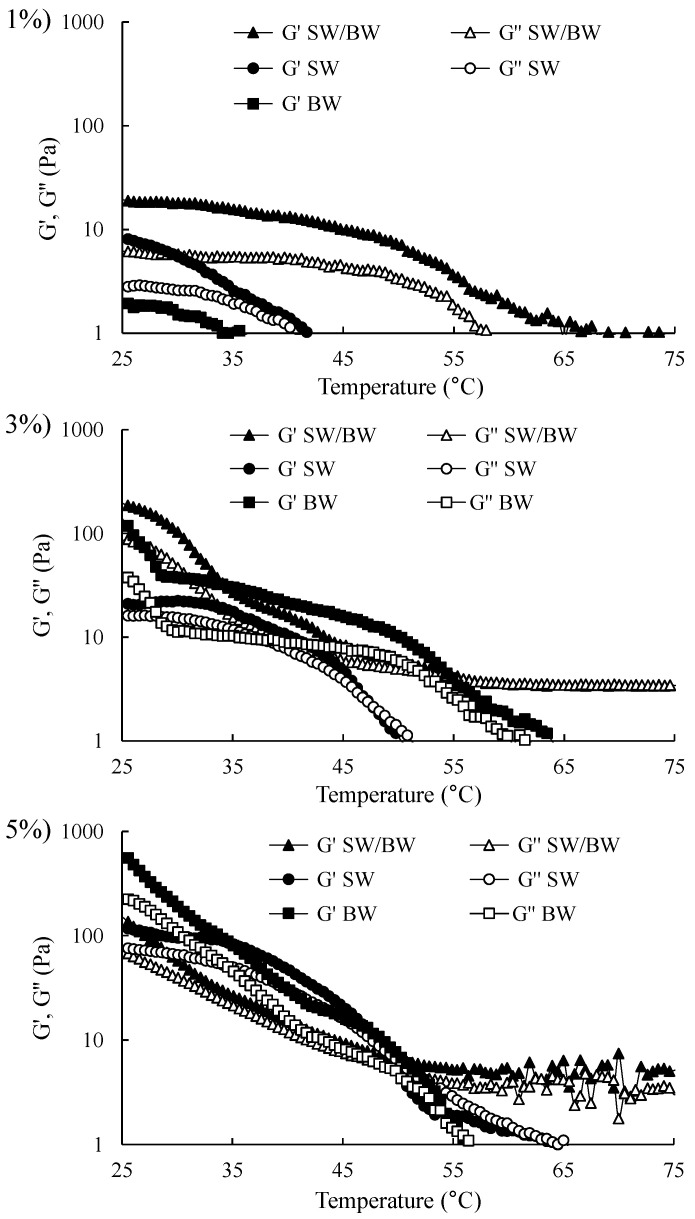
Temperature sweep in ChO structured with SW/BW, SW, and BW at 1, 3, and 5% oleogelant.

It is crucial to understand the thermal behaviour of structured ChO in the temperature ramp rheograms, which illustrate the changes in G′ and G″ during heating (from 25 to 100 °C) (Figure 5). The G′ and G″ moduli of all structured ChO at 1, 3, and 5% decreased with increasing temperature, indicating a gradual breakdown of the gel network. This breakdown is associated with reduced elastic properties and increased viscous properties of the structured oils [25]. The moduli tended to increase with higher levels of the structuring agent, with the highest G′ and G″ values observed at 5%. The results of the temperature sweep coincided with the microstructural observations (Figure 1), as oleogels with denser crystalline networks presented higher G′ values, which translated into slower destabilisation of the gel network. Furthermore, these trends were broadly consistent with the results obtained in the deformation and frequency tests (Figure 3 and Figure 4). SW and BW exhibited higher G′ values relative to G″ at temperatures at 25–50 °C and 25–55 °C, respectively, demonstrating dominant elastic properties (gel-like solid state). With further heating up to 100 °C, the gelled state was lost, leading to a crossover point where G″ equalled G′. Beyond this point, the gel transitioned entirely into a liquid state, and the structured ChO became viscous. In other studies, this behaviour has been reported in wax-structured oils [22,25]. The SW/BW also showed a decrease in both G′ and G″ with increasing temperature, indicating a weakening of the gel. However, a crossover point was observed around 65–70 °C in this region. As SW/BW consists of a mixed crystal system (SW and BW), the strength of the structured oil crystal lattice may have resulted in a more extensive melting temperature range in the rheogram [22].

#### 2.2.5. Thixotropic Properties

**Figure 6:**
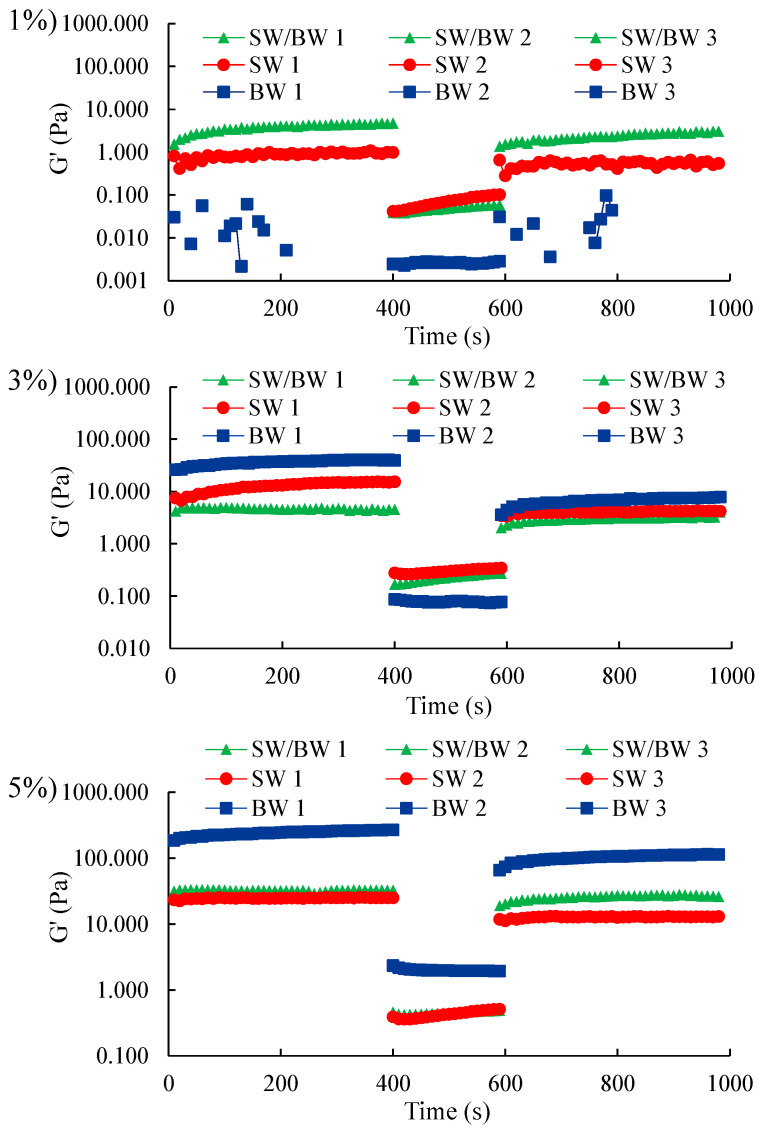
Thixotropic properties in ChO structured with SW/BW, SW, and BW at 1, 3, and 5% oleogelant.

Thixotropy was used to evaluate the structural recovery capacity of structured oil samples after applying a full deformation sweep [25]. The three-step oscillatory method was used to investigate the thixotropic behaviour of structured ChO subjected to low deformation (0.1%), very high deformation (100%), and then back to low deformation (0.1%), each for 400 s (Figure 6). 

In the time sweep measurements, SW showed superior elastic properties to BW and blend when the oleogelant concentration was 1%. Nevertheless, this trend was reversed when the oleogelant content was increased to 3% and 5%, with BW-structured chia oil exhibiting a more gelatinous behaviour than SW, which contributed to improving the elastic response of the blend. These differences were more noticeable with 5% oleogelant, attributed to the microstructural arrangement of the wax crystals, variations in crystal size and morphology between SW and BW (Figure 1), resulting in different crystalline structures in the resulting oleogels [44]. Thixotropic results revealed that ChO structured with a high oleogelant concentration of 3 and 5% maintained a more stable gelatinous structure, resistant to disruption under low deformation. When the stress amplitude was increased to 100% in the second sweep, with an oleogelant concentration of 1%, the ChO structured with the SW/BW blend and the SW control both retained superior elastic characteristics compared to the ChO structured with BW, which showed a significant alteration of the gelatinous network at this level of deformation. However, at 5%, BW showed behaviour dominated by strong elasticity. When the stress amplitude was increased to 100% in the second sweep, with an oleogelant concentration of 1%, the ChO structured with SW/BW blend and SW control retained superior elastic characteristics, whereas the oil structured with BW showed a significant alteration of the gelatinous network at this level of deformation. However, at 5%, BW showed behaviour dominated by strong elasticity.

A three-step time sweep was used to monitor thixotropic recovery. The gelatinous structure of the samples reformed as the deformation amplitude decreased within the linear viscoelastic region (LVR), indicating that the SW/BW-based structured ChO and the BW and SW controls exhibited thixotropic properties, characterized by partial structural recovery dependent on the oleogelant concentration. At an oleogelant concentration of 1%, when the deformation exceeded the maximum capacity of the network support structure, the BW gel support structure collapsed completely, rendering the samples more fluid and eliminating the observable thixotropic behaviour. This result is consistent with the findings reported by Hashemi et al. [45]. In contrast, an oleogelant concentration of 5% facilitated superior recovery in BW-based structured ChO, resulting in stronger, network-like gel structures. These observations are consistent with the viscoelasticity analysis. In particular, structured ChO prepared with SW and BW blend showed stable recovery responses across the entire range of oleogelant concentrations studied, even at low concentrations, suggesting a faster disruption of intermolecular interactions compared to their reassociation under shear conditions (Table S1, Supplementary Material). The results highlight the self-healing ability of double-network waxes with strong structural recovery. This phenomenon can be attributed to non-covalent interactions within the crystalline clusters and structural gel networks in the SW/BW samples [23]. The unique properties of oils with a double-network wax structure are highly relevant for applications requiring reversible structural decomposition and recovery, such as during food processing when used as a source of solid fat.

## 3. Conclusions

This study demonstrated that a slight increase in oleogelant concentration (1–5%) in the SW/BW blend significantly influenced the oil binding capacity (OBC) and firmness of the resulting oleogels. At 1%, limited crystal formation led to a weak structure with high oil migration. In contrast, at 3 and 5%, denser crystalline networks were formed, resulting in a semisolid texture with OBC values exceeding 75%. These findings suggest that the partial replacement of a single wax with a binary blend can produce an equally robust crystalline matrix, thereby enhancing the structural integrity and stability of ChO.

Furthermore, ChO structured with the SW/BW blend at 3 and 5% exhibited lower peroxide values (PV) than unstructured oil following the oleogelation process, underscoring the role of the semisolid matrix in limiting oxygen diffusion and delaying primary oxidation. Consequently, PV values remained below the maximum acceptable limit for edible fats and oils. Additionally, the ChO structured with the SW/BW blend exhibited a slightly more extended induction period compared to the unstructured ChO, indicating improved oxidative stability through the inhibition of lipid degradation reactions.

The viscoelastic behaviour of structured ChO prepared with 3 and 5% oleogelant concentrations exhibited a dominant semisolid gel character across a wide range of frequencies and temperatures. The SW/BW blend produced a more robust crystalline network, significantly enhancing the elastic properties and structural resistance to deformation, particularly at the 5% concentration. The study also showed that ChO structured with either SW or BW exhibited thixotropic behaviour, with structural recovery dependent on oleogelant concentration. Notably, oleogels prepared with the SW/BW blend demonstrated consistent and stable thixotropic recovery across all concentrations studied. These results highlight the potential of ChO structuring with the SW/BW blend at low concentrations as alternatives to fats in lipid-based foods such as emulsions, food fillings, and spreads, that require mechanical stability, elasticity, and resilience, as well as structural recovery after mechanical alteration during food processing.

## 4. Materials and Methods

### 4.1. Materials

Shellac wax (SW), SSB^®^ Cera 2, was acquired from SSB Stroever GmbH & Co. KG. (Bremen, Germany). Beeswax (BW) was purchased from Sigma Aldrich (St. Louis, MO, USA). Chia oil (ChO) was purchased from Aceitería Dumont (Santiago, Chile). All other chemicals used were of analytical grade.

### 4.2. Preparation of Structured ChO

The structured ChO was prepared using a SW/BW blend in a 1:1 ratio, using the individual waxes (SW and BW) as controls, by the methodology described by Morales et al. [24], with some modifications. The structuring process was carried out on a hot plate, using oleogelant concentrations of 1, 3, and 5% (*w*/*w*) at 82 °C for 30 min, with constant stirring at 300 rpm. After this time, the structured ChO was left to cool at room temperature for at least 2 h and then stored under refrigerated conditions (4 °C) and in the dark. After 24 h, the samples were analysed.

### 4.3. Oil Binding Capacity (OBC)

The oil binding capacity (OBC) of structured ChO with the SW/BW blend and controls was evaluated following the methodology described by Millao et al. [4]. The OBC test was centrifuged, subjecting the structured ChO to 7000 rpm for 40 min. Subsequently, 1 g of sample was weighed in 15 mL tubes. After centrifugation, the released oil was removed by inverting the tubes, and the remaining mass of the sample was recorded. The percentages of *OBC* and oil loss were calculated using Equation (1).

(1) $$OBC\;(\%)=\left(1-\left(\left(m_{i}-m_{f}\right)/m_{i}\right)\right)\times 100$$

where *m_i_* is the weight of the sample before centrifugation, and *m_f_* is the weight after the oil drainage.

### 4.4. Texture Analysis

The texture of the structured ChO with the SW/BW blend and controls was evaluated in terms of firmness, following the methodology described by Morales et al. [24]. The samples underwent a penetration test using the TA.XT PlusC texture analyser (Stable Micro Systems, Surrey, UK) equipped with a 5 kg load cell. Thirty grams of structured ChO were placed in a glass container and stored at 4 °C for 24 h. The samples were removed from storage immediately before testing. The analysis was conducted using a 5 mm cylindrical probe, which penetrated the sample within its container at a speed of 1 mm/s to a depth of 10 mm. The probe was then retracted to its original position at the same constant speed (1 mm/s). The maximum positive force, representing the firmness of the sample, was recorded and expressed in grams.

### 4.5. Confocal Laser Scanning and Optical Microscopy Analyses

The phase distribution of the structured ChO containing the SW/BW blend and the control samples was analysed using an Olympus FV1000 spectral confocal microscope (Olympus Corporation, Tokyo, Japan). Nile red (30 μM in acetone) was used to stain the oil phase, with excitation/emission wavelengths set at 488/590 nm. In addition, the microstructure of the structured ChO was examined by optical microscopy using a Euromex microscope (Euromex Microscope B.V., Diuve, The Netherlands) equipped with a 100× objective lens.

### 4.6. Fourier Transform Infrared (FTIR) Spectroscopy

FTIR analyses were conducted using an ATR-FTIR spectrometer (Cary 6390, Agilent, NC, USA). Samples were placed directly onto the diamond crystal of the ATR accessory and secured by applying gentle pressure to ensure proper contact. Measurements were carried out at room temperature, in a spectral range from 4000 to 600 cm^−1^, with a resolution of 4 cm^−1^ and 30 scans per sample. All analyses were performed in triplicate. The spectra obtained were processed using OriginPro 2019b software (OriginLab Corporation, Northampton, MA, USA), including baseline adjustment and normalization of relative transmittance.

### 4.7. Lipid Oxidation

The peroxide value (PV) was determined to assess the oxidation status of ChO structured with the SW/BW blend. The individual waxes (SW and BW) and unstructured ChO were also analysed as controls. The analysis was performed using the AOCS Cd 8–23 method. Samples (5 g) were treated with 30 mL of an acetic acid:chloroform solution (3:2 *v*/*v*), followed by the addition of 0.5 mL of a saturated potassium iodide solution. The liberated iodine was subsequently titrated with a 0.01 N sodium thiosulfate solution. The peroxide value (PV) was expressed as milliequivalents of peroxide per kilogram of sample and was determined in triplicate according to Equation (2):

(2) $$PV=\left(\left(S-B\right)\times M\times 1000\right)/W$$

where *S* is the volume of sodium thiosulfate solution used for the sample (mL), *B* is the volume used for the blank (mL), *M* is the molarity of the sodium thiosulfate solution (mol/L), and *W* is the weight of the sample (g).

### 4.8. Oxidative Stability

The oxidation test of structured ChO with the SW/BW blend and control samples was conducted in an OXITEST reactor (VELP Scientifica, Milan, Italy), following the procedure described by Chóez-Guaranda et al. [46]. For this purpose, 5 g of the sample was weighed and evenly distributed in the test cells. The device was set to a temperature of 90 °C and an oxygen pressure of 6 bar. Oxidative stability was determined using Oxisoft softwareversion 4.2.0 and expressed as induction period (IP) values.

### 4.9. Rheological Analysis

The viscoelastic properties of the samples were evaluated using a rheometer (Discovery DHR2, TA Instruments, New Castle, DE, USA) equipped with a rough parallel plate geometry (Plate SST ST XHatch, 40 mm Smart-SW) and a Peltier steel plate, with an axial gap of 1000 μm. The measurements were conducted following the method described by Morales et al. [24], with slight modifications. The TRIOS software package version 5.1.1 (TA 87 Instruments, New Castle, DE, USA) was used to operate the rheometer and acquire rheological data. Steady-shear flow measurements were conducted at 25 °C over a shear rate range of 1–1000 s^−1^. The linear viscoelastic region (LVR) of the samples was determined by plotting the storage modulus (G′) against oscillatory strain (%) under oscillatory conditions at 1 Hz, with strain ranging from 0.01% to 100%. In the frequency sweep test, the evolution of the storage (G′) and loss (G″) moduli was measured across a frequency range from 0.1 to 100 Hz at a constant strain of 0.1%, within the LVR. A temperature sweep test was performed to assess the thermal stability of the samples, from 20 to 90 °C, using a linear heating rate of 5 °C/min, at 0.1% strain and 1 Hz frequency. The three-step oscillation method to measure the thixotropic properties was conducted using three oscillation time sweeps with different strain amplitudes that are within and outside the LVR of the sample (400 s at a strain of 0.1%, 200 s at a strain of 100%, and 400 s at a strain of 0.1%). Three replicates of each sample were recorded for each test.

### 4.10. Statistical Analysis

A one-way analysis of variance (ANOVA) was performed with a significance level of 0.05. Results are reported as mean values ± standard deviation from replicate measurements. Post hoc comparisons were conducted using Tukey’s test to determine significant differences among treatments at *p* < 0.05. All statistical analyses were carried out using Minitab 19 software.

## Figures and Tables

**Table 1:** Physicochemical characterization of chia oil structured with the SW/BW blend and controls (SW and BW).

Samples	OBC (%)	Firmness (g)	PV (meq O_2_/Kg Sample)	IP_90 °C_ (h)
SW/BW 1%	43.6 ± 1.23 ^e^	nd	3.66 ± 0.23 ^bcd^	2.22 ± 0.01 ^abc^
SW/BW 3%	75.6 ± 1.12 ^c^	16.9 ± 0.37 ^d^	3.19 ± 0.20 ^abc^	2.24 ± 0.03 ^abc^
SW/BW 5%	88.4 ± 1.01 ^ab^	55.1 ± 4.15 ^b^	3.12 ± 0.20 ^ab^	2.38 ± 0.15 ^abc^
SW 1%	39.8 ± 0.71 ^f^	nd	3.86 ± 0.23 ^d^	1.93 ± 0.37 ^c^
SW 3%	67.3 ± 0.29 ^f^	9.59 ± 0.95 ^d^	3.85 ± 0.12 ^d^	1.87 ± 0.27 ^c^
SW 5%	87.6 ± 1.65 ^f^	31.6 ± 1.91 ^c^	3.79 ± 0.20 ^d^	2.19 ± 0.07 ^abc^
BW 1%	10.0 ± 1.24 ^g^	nd	3.45 ± 0.11 ^bcd^	2.22 ± 0.15 ^abc^
BW 3%	66.3 ± 1.04 ^d^	46.6 ± 2.89 ^b^	3.45 ± 0.30 ^bcd^	2.71 ± 0.31 ^ab^
BW 5%	91.3 ± 0.53 ^a^	134.8 ± 6.37 ^a^	2.73 ± 0.12 ^a^	2.79 ± 0.40 ^a^
ChO	−	−	3.71 ± 0.11 ^cd^	2.09 ± 0.09 ^bc^

Different letters in the same column represent significant differences determined by Tukey’s test (*p* < 0.05); nd: not detected.

## Data Availability

The original contributions presented in this study are included in the article/Supplementary Materials. Further inquiries can be directed to the corresponding author(s).

## Supplementary Materials

The following supporting information can be downloaded at: https://www.mdpi.com/article/10.3390/gels11090680/s1, Figure S1. FTIR spectra in ChO structured with SW/BW, SW, and BW at 1, 3, and 5 % oleogelant; Table S1. Recovery (%) in ChO structured with SW/BW, SW, and BW at 1, 3, and 5 % oleogelant [47].
