# Supplementary material for: Rheological and Physicochemical Characterization of Structured Chia Oil: A Novel Approach Using a Low-Content Shellac Wax/Beeswax Blend as Oleogelant
Source: Gels. 2025 Aug 25;11(9):680. doi: 10.3390/gels11090680 (PMC12469946; doi:10.3390/gels11090680)
Supplement: Supplementary file 1 [file gels-11-00680-s001.zip › gels-3783551-supplementary.pdf]

Supplementary material

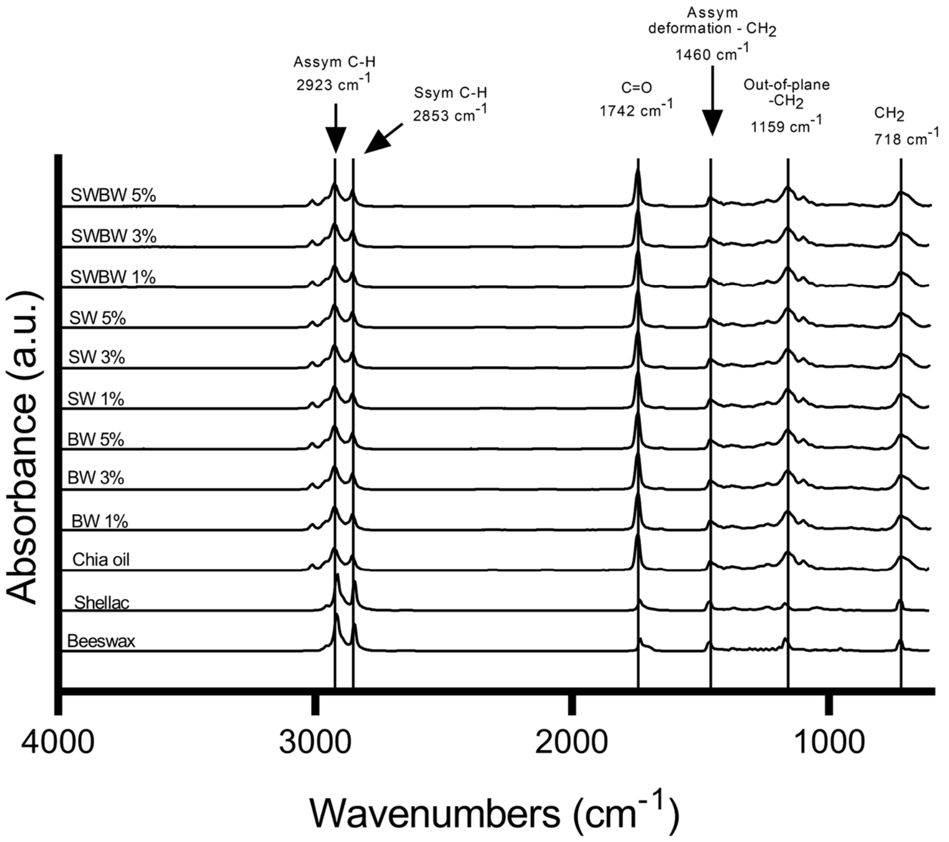

Figure S1. FTIR spectra in ChO structured with SW/BW, SW, and BW at 1, 3, and 5 % oleogelant.

Table S1. Recovery (%) in ChO structured with SW/BW, SW, and BW at 1, 3, and 5 % oleogelant.

ChO structured	G´ initial	G´50s	Recovery (%)
1% SW/BW	1.93 ± 0.57	2.00 ± 0.57	103.0 ± 4.36
1% SW	0.84 ± 0.19	0.39 ± 0.08	46.3 ± 0.93
1% BW	ND	ND	ND
3% SW/BW	3.63 ± 1.23	3.41± 0.61	96.6 ± 15.8
3% SW	13.0 ± 3.14	2.43± 0.00	19.2 ± 4.65
3% BW	3.25± 1.09	3.25 ± 0.79	69.7 ± 0.72
5% SW/BW	32.5 ± 0.41	19.4 ± 2.99	59.7 ± 8.45
5% SW	23.8 ± 1.91	9.06 ± 4.12	37.5 ± 14.3
5% BW	268.8 ± 4.39	73.7 ± 10.1	27.5 ± 4.21

. The recovery of the sample at 50 s after deformation of the samples can be determined by following equation: Recovery= $G'_i/G50$, where, G´i represents initial G′ value of the sample and G50 represents the G′ values of the samples within the first 50 s after deformation (Yilmaz et al., 2026). ND: Not determined

Yılmaz, M. T., Kutlu, G., Tulukcu, E., Toker, O. S., Sagdic, O., & Karaman, S. (2016). Rheological characteristics of Salvia sclarea seed gum solutions at different hydration temperature levels: Application of three interval thixotropy test (3ITT). LWT-Food Science and Technology, 71, 391-399.
